# Development of a core outcome set in the clinical trials of traditional Chinese medicine for diabetic foot: A study protocol

**DOI:** 10.3389/fmed.2022.1025833

**Published:** 2022-11-09

**Authors:** Xin Yue Dai, Ming Jie Zi, Chun Xiang Liu, Yi Ming Wang, Rui Gao

**Affiliations:** ^1^Institute of Clinical Pharmacology of Xiyuan Hospital, China Academy of Chinese Medical Sciences, Beijing, China; ^2^Evidence-Based Medicine Center, Tianjin University of Traditional Chinese Medicine, Tianjin, China

**Keywords:** diabetic foot, core outcome set, Chinese medicine, clinical trials, protocol

## Abstract

**Background:**

Diabetic Foot (DF) is one of the most common complications of diabetes, and it is characterized by high morbidity, disability, lethality and low cure rate. Traditional Chinese medicine treatment has certain characteristics and advantages in diabetic foot. Due to selective reporting bias and heterogeneity of research results, on the one hand, relevant clinical studies are of low quality and poor practicability, and on the other hand, similar studies cannot be included in meta-analysis to form high-quality evidence-based evidence and evaluate the value of studies. Therefore, the development of a core set of outcomes (COS-TCM-DF) for traditional Chinese medicine for the treatment of diabetic foot is an important way to address these issues.

**Methods:**

The COS-TCM-DF project will refer to the developed COS methodology and the steps are divided into four stages: (1) a scoping review and analysis of enrolled research protocols to collect and analyze all existing outcomes that have been used in trials on the prevention or treatment of diabetic foot with Chinese Medicine; (2) qualitative interviews with Diabetic foot patient and attendants to Collect additional outcomes related to them; (3) Gather opinionest and obtain initial consensus from key stakeholders, including patients, clinicians, researchers, and pharmaceutical company staff, through a Delphi survey; (4) a consensus meeting was held to develop the final COS-TCM-DF.

**Discussion:**

Currently, there is no COS on measuring or monitoring diabetic foot with TCM in trials or clinical practice. The COS-TCM-DF will be developed to advance the synthesis of evidence regarding the prevention and treatment of diabetic foot in TCM and to promote the standardized and consistent application of results in future studies in this field.

**Trial registration:**

Registered with the Core Outcome Measures in Effectiveness Trials (COMET) database, December 2019 https://www.comet-initiative.org/Studies/Details/1553.

## Introduction

Diabetic foot (DF) is the infection, ulceration, or destruction of tissues of the foot of a person with currently or previously diagnosed diabetes mellitus, usually accompanied by neuropathy and/or peripheral vascular disease (PAD) in the lower extremity (1). DF is one of the most common complications of diabetes, and it is characterized by high morbidity, disability, mortality, and adherence. It is estimated that every 20 s a person with diabetes is amputated in the world. (2, 3). It causes great pain and economic pressure to the patient, and also brings a heavy burden to the patient’s family, the medical industry and even society (4). Traditional Chinese medicine (TCM) has a variety of methods for the treatment of diabetic foot with obvious advantages, which can be divided into internal treatment and external treatment of TCM (5). External treatment of traditional Chinese medicine refers to the treatment of skin (mucous membrane) or from outside the body with drugs, manipulations, or instruments under the guidance of traditional Chinese medicine theory. It includes acupuncture, acupoint massage, traditional Chinese medicine foot bath and fumigation, etc., while the internal treatment of traditional Chinese medicine refers to oral Chinese herbal medicine or proprietary Chinese medicine. Modern research has shown that Chinese medicine treatment has significant effects on improving local blood circulation, promoting the regeneration of traumatic tissues and increasing the healing rate of trauma in diabetic foot patients. However, due to the quality limitation of existing Chinese medicine research, there is a lack of high-level evidence-based evidence, which restricts the application and promotion of Chinese medicine in the treatment of diabetic foot.

The selection of outcome indicators is particularly important for a study, which is closely linked to the quality and practicality of clinical studies. Recently, we have identified some problems by analyzing the outcome indicators in clinical studies of TCM for the treatment of diabetic foot: (1) TCM treatments for diabetic foot vary widely in the selection of RCT-reported outcome indicators, and the combination is arbitrary. (2) Lack of outcome indicators that reflect the characteristics and advantages of TCM. (3) The characteristics of TCM or significant endpoint indicators were insufficient. (4) The dimensions of outcome indicators are not comprehensive enough. It was urgent to establish the core outcome set of TCM in treating diabetic foot (6).

A core outcome set (COS) is a collection of the smallest and most important outcome indicators that should be measured and reported in a clinical trial for the same health domain (7). It aims to reduce the heterogeneity between the reported outcomes and strengthen the evidence synthesis value by lowering the risk bias of outcome reporting (8). The development of a core outcome set for traditional Chinese medicine in diabetic foot (COS-TCM-DF) will substantially reduce variation in reported outcomes, improve the quality and utility of clinical studies in Chinese medicine, and is important for international recognition of study results.

## Objectives

The purpose of this paper is to propose a protocol for the development of COS for a trial of Chinese medicine for the prevention or treatment of diabetic foot (COS-TCM-DF). We will reach a consensus about “what” to measure for diabetic foot with TCM in this study. We will further explore the “how” and “when” of measurement in future studies.

## Method

The COS-TCM-DF project has completed registration on the Core Outcome Measures in Effectiveness Trials initiative (COMET) website (9). The present study protocol was written in accordance with a series of standards (10–13) formulated by the COMET group. The scope of this COS-TCM-DF is:

• Health condition: diabetic foot
• Target population: diabetic foot patients without any age or sex restrictions
• Intervention: traditional Chinese medicine treatment
• Settings: clinical trials, other types of clinical research, and System evaluation

**FIGURE 1:**
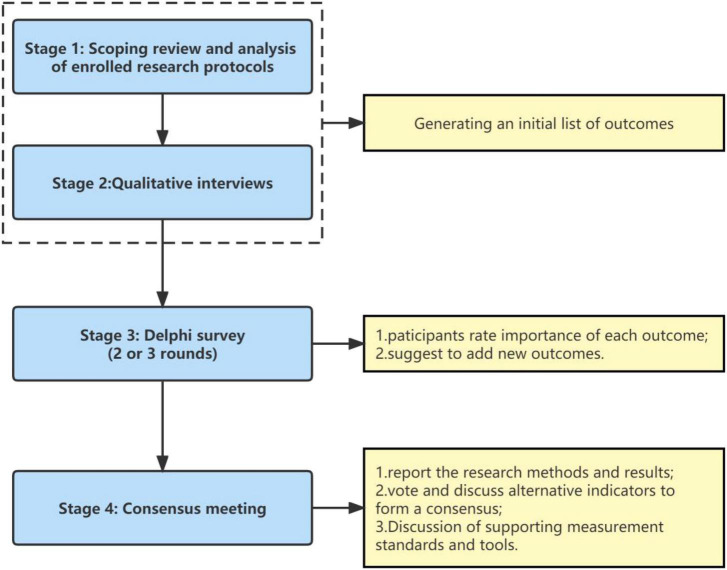
Key phases in process.

The development of COS-TCM-DF will include four phases: a scoping review and analysis of the existing results of the registration study protocol, qualitative interviews, 2 to 3 rounds Delphi surveys, and a consensus meeting (Figure 1).

## Stage 1: Scoping review and analysis of enrolled research protocols

To capture what to measure and to identify the existing knowledge, we will start a scoping review of all outcomes used in published clinical trials and research protocols which Registered on Chinese clinical trial registration website (14) and American clinical Study registration website (15) regarding the prevention and treatment of diabetic foot with TCM and develop an inclusive list of the outcomes. The study is divided into the following two parts:

## Part I: Systematic evaluation of traditional Chinese medicine treatment of diabetic foot

### Search strategy

We will search five databases: MEDL INE (*via* PubMed), Embase, CNKI, Wan Fang Data and the Cochrane Central Register of Controlled Trials (CENTRAL) in this review. The search date is limited to the period between the completion of the database and 2020.4.30. In our search strategy, we will use both free-text and subject headings based on the following main concepts: diabetes foot OR diabetic feet, diabetic foot, traditional Chinese medicine, TCM, RCT. Based on the electronic search, we will further hand search references cited in those identified eligible studies and relevant systematic reviews.

### Eligibility criteria

The inclusion and exclusion criteria are shown in Table 1.

**TABLE 1:** The inclusion and exclusion criteria for systematic reviews.

Inclusion criteria	Exclusion criteria
Patients with diabetic foot	Patients with other complications
TCM (Chinese herbs, herbal decoctions, Chinese patent medicine (CPM) and acupuncture) or TCM related therapies	Not a TCM intervention
Randomized controlled trials	Full-text cannot be obtained
Standardized writing, with clear criteria for diagnosis, inclusion and exclusion, and efficacy evaluation	Not published in Chinese or English

### Study selection and data extraction

Literature screening and data extraction will be performed independently by two researchers with strict reference to inclusion and exclusion criteria, and cross-check. Data will be extracted and recorded on a pre-defined form. Any disagreements that arise during the process will be addressed through discussion and consensus with the study team. The extracted information included title, author, sample size, intervention, outcome indicators, measurement method, etc.

## Part II: Analysis of enrolled research protocols

### Search strategy

We will search the Chinese clinical trial registration website (14) and American clinical Study registration website (15) to obtain clinical trials of TCM for the treatment of diabetic foot. The search date is limited to the period between the completion of the database and 2021. The search terms are “diabetic foot”, “diabetic foot ulcer”, “diabetes foot”, “diabetic foot”, etc.

### Eligibility criteria

The inclusion and exclusion criteria are shown in Table 2.

**TABLE 2:** The inclusion and exclusion criteria for enrolled research protocols.

Inclusion criteria	Exclusion criteria
Clinical studies related to diabetic foot	Survey and Research on Evidenceology
TCM [Chinese herbs, herbal decoctions, Chinese patent medicine (CPM) and acupuncture] or TCM related therapies	

### Study selection and data extraction

Two investigators independently read the registration information, excluded studies that did not meet the criteria based on the screening criteria, and cross-checked. For the included studies two investigators extracted the information independently, and any disagreement during the process was decided through discussion. Data were extracted using a pre-designed form, and information collected included: enrollment number, study title, study design, enrollment time, study population, intervention, outcome indicators, and duration of treatment.

### Data analysis

Key information from the included literature and registered studies will be presented in tables and descriptively. Outcomes with the same meaning but different presentation will be combined into one standard outcome after data extraction.

## Stage 2: Qualitative interviews

After completing the literature study, we will conduct qualitative interviews with diabetic foot patients and caregivers to obtain supplementary indicators from their perspectives in order to supplement the item pool. The qualitative interviews is aimed to get the perspectives of patients and caregivers on the outcome indicators of TCM treatment of diabetic foot, and to avoid missing any important outcomes (10, 16).

### Participant selection

In this study, semi-structured interviews were conducted with diabetic foot patients and caregivers. The inclusion and exclusion criteria for the semi-structured interviews are shown in Table 3.

**TABLE 3:** The inclusion and exclusion criteria for the semi-structured interviews.

Inclusion criteria	Exclusion criteria
Patients with diabetic foot (age ≥18 years)	Patients and caregivers who are unable to communicate properly
Caregivers who are taking care of patients with diabetic foot	
Patients/caregivers voluntarily participated and oral informed consent	

### Sampling strategy

The aim of the semi-structured interviews is to achieve “data saturation” (17). Data collection for the interviews will be terminated when data saturation occurs, which is defined as no generation of new themes and outcomes. We will continue interviewing two more participants when no new themes or outcomes emerge to confirm the saturation of data (18, 19).

### Preparation and data collection

According to the research purpose and early results, an interview outline is formed. Before the interview, the interviewer shall be trained uniformly to ensure that the interviewer is familiar with the interview content and improve the accuracy and scientifically of communication. The training content includes the interpretation of interview outline, the determination of the questioning process, questioning techniques and methods, matters needing attention and so on. Prior to the interview, informed consent of the interviewee should be obtained. The outline of the semi-structured interviews is shown in Table 4.

**TABLE 4:** The outline of the semi-structured interviews.

Patients	Caregivers
When were you diagnosed with a diabetic foot?	When was the patient you are accompanying diagnosed with a diabetic foot?
What inconvenience or discomfort did you suffer from a diabetic foot?	What do you think are the inconveniences of taking care of diabetic foot patients?
What therapeutic effects do you hope to achieve?	What therapeutic effects do you hope to achieve for the patient you are accompanying?
What are the outcomes that you are most concerned about?	What are the outcomes that you are most concerned about?

### Data analyses

We will transcribe and analyze the content of the interviews. In order to protect the privacy of the interviewees, we will adopt the form of anonymity and encrypt all interview materials. The recordings were encoded and analyzed by two researchers and then checked against each other. Any disagreements during the process will be decided by the team through discussion. Finally, the interview results of diabetic foot patients and caregivers were summarized and added to the original item pool for further research.

## Stage 3: Delphi survey

The results of literature research and qualitative interview form the original item pool. Through group discussion, standardize the entry pool, including merging synonyms, splitting compound entries, etc. Electronic questionnaires will be developed after the specification of the items is completed. The questionnaire is divided into four parts: (1) introduce the background, purpose, significance and time of this study; (2) Participants’ basic information collection: such as region, title, working years and major; (3) To score the importance of the outcome index, add an open question at the end of the score, and the participants put forward suggestions for modification and supplement and explain the reasons; (4) Participants’ self-assessment. The Delphi survey is a technique for reaching a consensus on a topic by gathering the opinions of relevant stakeholders (20). It is often used in the research of development of COS. The Delphi survey will run two or three rounds until a consensus is reached. After each round, the survey results will be fed back to the participants. The goal is to facilitate the generation of consensus on the most important outcomes through an iterative process (20).

### Stakeholder selection

Participants in the Delphi survey included four stakeholder groups: (1) Diabetic foot patients and caregivers; (2) professionals in peripheral vascular surgery or endocrinology; (3) Pharmaceutical company researchers; (4) methodologists. This study will aim to recruit about 100 participants.

### Process

To avoid ambiguity, the descriptions in the questionnaire should provide both medical terminology and layman’s language (12). In order to optimize questionnaire design and language expression, representatives of each stakeholder groups will be chosen for pre-test before the study is formally carried out. The optimized questionnaire makes it easier for participants to better understand the content of Delphi survey, so as to reduce the deviation.

In order to reduce the rate of lost follow-up, we will send electronic questionnaires to potential interviewees before the study to confirm that they can participate in and complete two or three rounds of the survey. After confirmation, we will send links to the questionnaire *via* email or WeChat. Respondents who did not complete the survey in the first round will be excluded from the next round of the survey. In each round of the survey, we will send weekly emails to participants who have not submit to ensure the completion rate. It will last for 3 weeks during each Round of Delphi survey. If the response rate is too low for two rounds, the survey will continue to open for 1–2 weeks to reduce potential attrition bias.

The GRADE panel’s recommended scale of 1–9 will be used to rate the importance of each candidate outcome, with 1–3 for limited importance, 4–6 for important but not critical, and 7–9 for critical (21). We will conduct a statistical analysis of the results at the end of each round. The open question answers were extracted by two researchers, respectively. For ambiguous answers, participants were asked to clarify. Only candidate indicators which more than 70% of respondents scored 7–9 were included in the second round of the survey. Participants’ supplementary indicators were discussed by the research group to decide whether to include them in the next round of questionnaires. The results of each round of questionnaire and their own score in that round will be fed back to each participant at the beginning of the next round of the questionnaire.

## Stage 4: Consensus meeting

After the candidate of COS is determined through Delphi investigation, senior representatives of different participating groups should be convened to reach a consensus through discussion and determine the final COS.

### Participants

Participants of the consensus meeting included representatives of various interest groups who completed the Delphi survey, members of the working group and representatives of senior experts who had not participated in the research process.

### Process

Consensus meetings will be held face-to-face. In exceptional cases, a video conference can be held online. The principal investigator clearly reported the process of Delphi investigation and identified COS candidate outcomes to the conference participants. The participants of different groups will vote before full discussion. Finally, a consensus was reached after further discussion based on the voting results. If there is a difference of opinion during the process, the nominal group method is adopted to resolve it.

The contents of the consensus meeting include: (1) report on the research methods and results of COS-TCM-DF; (2) Invite experts to vote and discuss alternative indicators to form a consensus; (3) Discuss whether corresponding measurement tools and methods can be recommended for the included outcome indicators.

### Consensus definition

The consensus criteria is more than 70% of the respondents score 7–9 (22).

## Dissemination

After the research is completed, we will publish the COS-TCM-DF through industry associations and COMET platforms. And through academic conferences and papers published to promote the application. We will engage with relevant groups/organizations. We will also provide a summary of the results to all participants.

## Discussion

Traditional Chinese medicine plays an important role in the healthcare system as a complementary and alternative medicine (23). Traditional Chinese medicine treatment has certain characteristics and advantages in diabetic foot. A meta-analysis study showed that TCMIs (Traditional Chinese Medicine Injections) can increase the clinical effective rate of conventional therapies by 27%. Along with a better performance in safety and financial burden, the management of DF can be improved by TCMIs (24). Numerous studies have also confirmed the efficacy of other TCM treatments. However, due to the quality limitation of existing Chinese medicine research, there is a lack of high-level evidence-based evidence, which restricts the application and promotion of Chinese medicine in the treatment of diabetic foot. One of the important reasons for the low quality of studies is that there is no appropriate standardized outcome index. The selection of outcome indicators is particularly important for study quality. It is essential in the synthesis and conversion of evidence to use COS (25). A COS for clinical trials regarding the traditional Chinese medicine treatment of diabetic foot will be developed by the COS-TCM-DF study. The research plan of strictly follows the best method guidelines provided by the COMET initiative [COS-STAD minimum standard (12) and COS-STAP statement (11)], learning from COS protocols that have been published in other fields. The development of COS-TCM-DF will standardize the selection of relevant clinical research indicators and improve the quality of clinical research. The COS-TCM-DF will be applied to all clinical studies of diabetic foot with TCM interventions. We expect to recommend common outcome indicators for diabetic foot as a whole, and may also have specific indicators for different stages of the disease. This COS will significantly improve the standardization of reporting of relevant study outcome indicators and reduce the heterogeneity of reporting of similar study outcome indicators. Therefore, the conduct of our study is very meaningful. However, in the literature research part of this study, the language is limited to English and Chinese. Other aspects of the literature language were not included, which could create potential bias. We will publish the study in a journal to facilitate communication when it is completed.

## Ethics statement

The Ethics Committee of Xiyuan Hospital of CACMS has approved and passed the project (2020XL013-2). Informed consent was obtained from patients participating in semi-structured interviews, Delphi surveys, and consensus meetings.

## Author contributions

XD and RG conceived this study. XD drafted this manuscript. XD, RG, MZ, CL, and YW participated in the study design process of the manuscript. All authors agreed to submit the final manuscript after a rigorous and systematic review of the manuscript.

## Abbreviations

DF: diabetic foot
COS: core outcome set
COS-TCM-DF: core outcome set of traditional Chinese medicine for diabetic foot
TCM: traditional Chinese Medicine
COMET: core outcome measures in effectiveness trials
COS-STAP: core outcome set-STAndardised protocol
COS-STAD: core outcome set-STAandards for Development.
